# The promotion of non-treatment physical activity in physiotherapy and exercise physiology practice in an Australian regional hospital: A mixed-methods study

**DOI:** 10.1016/j.jsampl.2023.100020

**Published:** 2023-01-16

**Authors:** Stephen Barrett, Kane Rodda, Owen Howlett, Alistair Mumford, Donna Borkowski, Josh Naunton, Stephen Begg, Breanne Kunstler, Marcos De Noronha, Michael Kingsley

**Affiliations:** a Research and Innovation, Bendigo Health Care Group, Victoria, 3552, Australia; b Holsworth Research Initiative, La Trobe University, Bendigo, Victoria, Australia, 3552; c Outpatient Rehabilitation Services, Bendigo Health Care Group, Victoria, 3552, Australia; d Physiotherapy Department. Bendigo Health Care Group, Victoria, 3552, Australia; e Physiotherapy Department, School of Primary and Allied Health Care, Faculty of Medicine, Nursing and Health Sciences, Monash University; f Violet Vines Marshman Centre for Rural Health Research, La Trobe Rural Health School, Bendigo, Victoria, Australia; g BehaviourWorks Australia, Monash University, Melbourne, Australia; h La Trobe Rural Health School, Bendigo, Victoria, Australia; i Department of Exercise Sciences, University of Auckland, New Zealand

**Keywords:** Physical therapy, Health promotion, Rural health, Exercise, Experiences

## Abstract

**Objectives:**

To describe the frequency of non-treatment physical activity (NTPA) promotion by physiotherapists and accredited exercise physiologists (AEPs) practicing in an Australian regional hospital. To identify factors that influence whether physiotherapists and AEPs promote NTPA to hospital patients.

**Design:**

Mixed-methods using sequential explanatory design.

**Methods:**

Physiotherapists and AEPs working in a regional hospital setting were invited to complete an online clinician survey and participate in semi-structured interviews. Likert scale questions measured frequency and factors influencing NTPA promotion. Qualitative descriptive analysis was used to analyse interview data.

**Results:**

In total, 41 clinicians completed the survey. Of the survey respondents, 66% reported always or often promoting NTPA to their patients. In outpatient settings, 72% of respondents always or often promoted NTPA compared to 54% in inpatient settings. Survey respondents reported high levels of knowledge and confidence in NTPA promotion. Confidence promoting NTPA with unmotivated patients was 54%. Eleven clinicians participated in the interviews. The interviews identified three themes that influenced NTPA promotion in hospital practice: (1) clinicians prioritise addressing the presenting condition before NTPA; (2) clinicians believe that the patients’ motivation influences NTPA promotion; and (3) clinicians rely on their professional and interpersonal skills, and exposure to appropriate training, to promote NTPA.

**Conclusion:**

Physiotherapists and AEPs frequently promote NTPA, especially in outpatient settings. The results suggest that low patient motivation to be active, the need to prioritise hospital discharge criteria before NTPA promotion and having few resources guiding NTPA promotion might negatively impact clinicians’ ability to promote NTPA as often as they would like.

## 1 Introduction

Insufficient physical activity (PA) is a major public health issue [1]. Insufficient PA is a leading modifiable risk factor for non-communicable diseases and the fourth biggest cause of mortality worldwide [2]. Individuals living in rural and regional areas have a higher prevalence of insufficient PA compared with metropolitan residents [3]. Insufficient PA results in a large economic burden on society, with individuals who undertake insufficient PA consuming more healthcare resources than those who are physically active [4]. Due to the associated burden on healthcare systems, clinicians are encouraged to integrate PA promotion into routine care and to use every clinical consultation as an opportunity to promote PA [5].

Physiotherapists and accredited exercise physiologists (AEPs) are suitably qualified and sufficiently skilled to help patients improve their PA [6]. Physiotherapists and AEPs practicing in hospital settings largely work with people with chronic conditions and competently employ PA as a treatment modality [6,7]. Promoting PA to improve and maintain general health is distinct from the utilisation of PA to rehabilitate an impairment and is called non-treatment physical activity (NTPA) promotion [6].

Physiotherapists largely consider the provision of NTPA advice to patients as part of their role, but many find it difficult to integrate NTPA into practice [8,9]. Lowe and colleagues found that 77% of UK-based physiotherapists routinely discussed PA with patients [10], though lower rates were identified amongst physiotherapists sampled in Australia (43%) [11] and America (41%) [12]. Patients believe that physiotherapists should promote NTPA alongside specific physical interventions [13]. These data, combined with high prevalence of insufficient PA worldwide indicate that many clinicians are missing an important opportunity to promote health enhancing behaviours such as PA.

Non-treatment physical activity promotion within routine clinical care is an increasing area of clinical interest and research [5]. Although the extant literature specific to NTPA promotion by physiotherapists is growing [5,11,12,14], studies investigating the promotion of NTPA by AEPs are scarce. In addition, relatively little is known about the extent to which physiotherapists and AEPs working in regional public hospital settings incorporate NTPA promotion into their clinical practice and what factors influence their engagement in NTPA promotion. As such, the aim of this study was to investigate the frequency and determinants of NTPA promotion by physiotherapists and AEPs practicing in inpatient and outpatient settings in an Australian regional public hospital. These data might offer distinctive perspectives on the implementation of health promotion into routine hospital care.

The objectives for this mixed-methods study were to.

1. Describe the frequency of NTPA promotion by physiotherapists and AEPs practicing in inpatient and outpatient settings in an Australian regional public hospital.
2. Identify factors that influence whether physiotherapists and AEPs promote NTPA to hospital patients.

## 2 Methods

A mixed-methods study was conducted using a sequential explanatory design [15]. This was a two-stage process, beginning with the self-reported clinician survey investigating current practice in NTPA promotion. The second stage involved the collection and analysis of in-depth interviews to gain insight into behaviours, beliefs and attitudes of physiotherapists and AEPs towards NTPA promotion in public hospital practice. The integration of methods is highlighted in Supplement A. Ethical approval was obtained from the Bendigo Health Care Group Ethics Committee and the La Trobe University Human Research Ethics Committee (reference number: LNR/72566/BH-2021-253248). All participants gave informed consent before data collection began.

All physiotherapists and AEPs practicing full- or part-time in inpatient and outpatient settings in a major tertiary hospital in regional Australia were eligible to participate. Participation was offered to all physiotherapists (n ​= ​95) and AEPs (n ​= ​11) who were consulting with patients between April 2021 and May 2021. During the period when the survey was open the ratio of inpatient to outpatient clinicians was 2:1.

For the clinician survey, an email containing the study information and the survey link was sent to the physiotherapists and AEPs in the participating hospital by the Chief Allied Health Officer. Reminder emails were sent at two-weeks and four-weeks following the initial email. For the clinician interviews, participants were asked at the beginning of the survey if they were interested in taking part in a semi-structured interview. Willing individuals were contacted via email to arrange an interview. All participants provided written informed consent prior to commencing the interview.

Participants completed a self-administered survey that collected demographic information, frequency of NTPA promotion, and factors influencing their choice to promote NTPA or not (Supplement B). This was based on 18 behavioural domains, including the 14 domains from the theoretical domains framework (TDF) [16]. These questions covered the main domains (e.g., intentions; beliefs about capabilities) of behaviour, in this instance the promotion of NTPA. Responses were measured using 5-point Likert scales (i.e., strongly disagree, which was anchored to number 1, up to strongly agree, anchored to number 5). The survey was based on prior research investigating physiotherapists’ choice to promote NTPA to patients with musculoskeletal conditions [11]. The survey was adapted for use with hospital clinicians and pilot-tested with 8 purposively selected physiotherapists and AEPs to assess clarity, consistency, relevance to practice and face validity. The clinicians were purposively selected based on age, gender, and area of clinical practice. Based on feedback we provided an example of NTPA promotion in clinical practice at the start of the survey, and added the definition of NTPA to each new section. Clinicians involved in survey piloting were eligible to participate in the study. Surveys were administered via a secure online platform (QuestionPro, Texas, USA).

Employing a building approach to mixed-methods integration, results from the quantitative analyses informed the interview questions. Three hospital clinicians (two physiotherapists; 1 AEP) were consulted on the development of the interview guide (Supplement C). These three clinicians were chosen as they worked in inpatient, outpatients and community settings. These clinicians did not take part in the interviews. All interviews were carried out in-person and were led by authors SB and AM. Neither interviewer had a personal relationship or professional oversight over any of the interview participants. Interviews were conducted at the participating hospital at a time and place convenient to participants. All interviews were recorded with participants’ permission. Recordings were first transcribed using an online provider (Rev, Texas, USA) and then checked by SB. Participants were provided with fully checked transcripts for verification. Verified transcripts were used in the analysis. Field notes were used to supplement the audio and transcripts and informed the iterative development of interview guides and question-related probes for subsequent interviews.

Survey data were exported into SPSS (Version 28.0; IBM Corp., US) for analysis. Participants were not obliged to answer all questions; partial responses were included. Descriptive statistics, including frequencies (percentages) and means and standard deviations were used to summarise survey data.

Data from in-depth interviews were collected and analysed concurrently. Qualitative description was used as the theoretical framework for the qualitative analysis [17]. Qualitative description provides comprehensive straightforward descriptions of experiences in a language similar to the participant's own. Transcripts were analysed and coded line-by-line using Nvivo (Version 12; QSR International, Cambridge, MA, USA). Two transcripts were independently coded by three researchers (SB, KR and OH) to inform the development of a codebook. SB coded all transcripts; KR and OH coded 7 transcripts each. Categories and themes were identified and refined by SB, KR and OH until final themes were agreed by all authors. The number of new codes that were generated decreased with time; interviews 10 and 11 produced four and one new codes respectively, after which additional interviewing was ceased.

Michie and colleagues identified three components that influence behaviour (B): capability (C), opportunity (O) and motivation (M); together these components make up the COM-B model [18]. After data were coded into themes, two doctoral researchers (SB and OH) mapped identified themes to the domains of the COM-B model [18]. Both researchers have undertaken training in behaviour change technique (BCT) coding and have experience of mapping themes to the COM-B model. Where consensus was not reached, differences were resolved by discussion. Three authors (SB, OH and KR) mapped the consensus categories to the COM-B intervention functions [18], which guided our recommendations for practice and policy.

In line with a contiguous narrative approach to mixed-methods integration, the results of the quantitative and qualitative components are reported separately [19].

## 3 Results

Forty-one respondents completed the survey (response rate of 39%) and interviews were carried out with 11 clinicians. The demographics of survey respondents and interview participants are detailed in Table 1. Most respondents were female, aged between 25 and 34 years working in outpatient settings.

**Table 1:** Demographic characteristics of the survey and interview respondents.

	Survey		Interview	
Characteristic	Number	%	Number	%
Profession (n ​= ​41)				
AEP	9	22%	2	18%
Physiotherapist	32	78%	9	72%
**Gender (n** ​**=** ​**41)**				
Male	17	41%	6	55%
Female	24	57%	5	45%
**Age (n** ​**=** ​**41)**				
18–24	5	12%	–	–
25–34	22	52%	–	–
35–44	8	19%	–	–
45–54	4	10%	–	–
55–64	1	2%	–	–
Prefer not to answer	2	5%	–	–
**Year of experience (n** ​**=** ​**41)**				
0–2 years	4	10%	2	18%
3–5 years	9	22%	3	27%
6–10 years	12	29%	5	46%
11–15 years	6	15%	0	0%
16 ​+ ​years	10	24%	1	9%
**Healthcare setting (n** ​**=** ​**41)**				
Inpatient - acute	8	19%	1	9%
Inpatient - rehab	6	15%	1	9%
Outpatient - clinic setting	18	44%	6	55%
Outpatient - community setting (including home visits)	9	22%	3	27%
**Additional education with psychology focus (n** ​**=** ​**40)**				
Yes	16	40%	–	–
No	24	60%	–	–
**Additional education with health promotion focus (n** ​**=** ​**40)**				
Yes	13	32%	–	–
No	27	68%	–	–

The survey results demonstrated that 66% of respondents reported often or always promoting NTPA to their patients (Table 2). Seventy-eight percent of respondents often or always provided verbal NTPA advice to patients. In total, 59% of respondents never or rarely provided patients with written information on NTPA and 68% never or rarely referred patients to additional services to promote NTPA. Analysis by setting indicated that 72% of clinicians practicing in outpatient settings often or always promoted NTPA compared to 54% in inpatient settings. Higher rates of NTPA promotion were reported by AEPs (100% often or always promote NTPA and 100% often or always provide verbal advice) compared with physiotherapists (55% and 72% respectively).

**Table 2:** Rates of non-treatment physical activity promotion by physiotherapists and accredited exercise physiologists practicing in a public hospital setting.

Question	Responses				
**How often did you encourage your patients to have a more physically active lifestyle specifically for general health purposes?**	Never	Rarely	Sometimes	Often	All of the time
All clinicians (n ​= ​41)	–	7%	27%	**34%**	32%
Physiotherapists (n ​= ​32)	–	9%	**34%**	35%	22%
AEPs (n ​= ​9)	–	–	–	33%	**67%**
Clinicians practicing in outpatient settings (n ​= ​28)	–	3%	25%	**36%**	**36%**
Clinicians practicing in inpatient settings (n ​= ​13)	–	16%	**31%**	**31%**	23%
Additional training psychology focus (n ​= ​16)	–	12%	19%	31%	**38%**
Additional training health promotion focus (n ​= ​13)	–	16%	**31%**	**31%**	23%
≤5 years of clinician experience (n ​= ​13)	–	8%	23%	**38%**	31%
6–10 years of clinician experience (n ​= ​12)	–	–	25%	33%	**42%**
≥11 years of clinician experience (n ​= ​16)	–	12%	**32%**	**32%**	25%
**What percentage of patients did you provide verbal NTPA advice to?**	None	1-25%	26-50%	51-75%	>75%
All clinicians (n ​= ​41)	–	15%	7%	24%	**54%**
Physiotherapists (n ​= ​32)	–	19%	9%	31%	**41%**
AEPs (n ​= ​9)	–	–	–	–	**100%**
Clinicians practicing in outpatient settings (n ​= ​28)	–	18%	7%	18%	**57%**
Clinicians practicing in inpatient settings (n ​= ​13)	–	8%	8%	38%	**46%**
Additional training psychology focus (n ​= ​16)	–	19%	19%	–	**62%**
Additional training health promotion focus (n ​= ​13)	–	8%	8%	38%	**46%**
≤5 years of clinician experience (n ​= ​13)	–	**-**	9%	38%	**53%**
6–10 years of clinician experience (n ​= ​12)	–	–	8%	38%	**46%**
≥11 years of clinician experience (n ​= ​16)	–	21%	5%	18%	**56%**
**What percentage of these patients did you provide written NTPA advice (e.g. pamphlet, summary sheet) ​to?**	None	1-25%	26-50%	51-75%	>75%
All clinicians (n ​= ​41)	22%	**37%**	17%	19%	5%
Physiotherapists (n ​= ​32)	25%	**34%**	13%	25%	3%
AEPs (n ​= ​9)	11%	**45%**	33%	–	11%
Clinicians practicing in outpatient settings (n ​= ​28)	21%	**33%**	21%	18%	7%
Clinicians practicing in inpatient settings (n ​= ​13)	23%	**46%**	8%	23%	–
Additional training psychology focus (n ​= ​16)	13%	**43%**	25%	13%	6%
Additional training health promotion focus (n ​= ​13)	23%	**46%**	8%	23%	–
≤5 years of clinician experience (n ​= ​13)	24%	31%	7%	**38%**	–
6–10 years of clinician experience (n ​= ​12)	16%	25%	**41%**	18%	–
≥11 years of clinician experience (n ​= ​16)	19%	**38%**	19%	12%	12%
**What percentage of patients did you refer to other service providers/agencies for NTPA support (e.g. strength training groups, walking groups)?**	None	1-25%	26-50%	51-75%	>75%
All clinicians (n ​= ​41)	24%	**44%**	20%	5%	7%
Physiotherapists (n ​= ​32)	28%	**41%**	19%	6%	6%
AEPs (n ​= ​9)	11%	**56%**	22%	–	11%
Clinicians practicing in outpatient settings (n ​= ​28)	18%	**46%**	25%	4%	7%
Clinicians practicing in inpatient settings (n ​= ​13)	**38%**	**38%**	8%	8%	8%
Additional training psychology focus (n ​= ​16)	13%	**50%**	18%	6%	13%
Additional training health promotion focus (n ​= ​13)	36%	**46%**	15%	**-**	3%
≤5 years of clinician experience (n ​= ​13)	23%	**38%**	18%	14%	7%
6–10 years of clinician experience (n ​= ​12)	25%	**33%**	18%	16%	8%
≥11 years of clinician experience (n ​= ​16)	32%	**56%**	**-**	6%	6%

AEP: Accredited exercise physiologist; NTPA: Non-treatment physical activity.

Responses to all survey questions are detailed in Supplement D. The majority of respondents (87%) agreed that it is their responsibility as a physiotherapist or AEP to promote NTPA. Knowledge of how to promote NTPA was high (90%), and almost all respondents (97%) were confident to promote NTPA; however, confidence decreased to 54% for all respondents when they perceive patients as unmotivated. Respondents reported favourable beliefs about the consequences of NTPA promotion, with 97% believing NTPA promotion is worthwhile and 91% believing it would help patients become more active. Sixty percent of all respondents felt that patients’ presenting condition was a higher priority than NTPA promotion. Survey responses highlighted a number of barriers to NTPA promotion, including low patient motivation (37%), inadequate workplace resources (54%) and training (49%). The majority of respondents reported that workplace management were willing to listen (75%) and could be counted on for support (78%) in promoting NTPA.

Three themes were identified from the qualitative analysis of physiotherapists and AEPs’ attitudes and beliefs towards NTPA promotion in hospital practice: (1) clinicians prioritise addressing the presenting condition before NTPA; (2) clinicians believe that patient motivation influences NTPA promotion; and (3) clinicians rely on their professional and interpersonal skills, and exposure to appropriate training, to promote NTPA. The codes and categories of the corresponding themes are detailed in Supplement E; described below are verbatim quotes to illustrate and substantiate the themes. Additional verbatim quotes for each theme are provided in Supplement F. Quotes are identified by profession and their work setting.

### 3.1 Theme 1: Clinicians prioritise addressing the presenting condition before NTPA

Decisions to promote NTPA were heavily influenced by clinical priorities, which appeared to be set organisationally and individually. For clinicians in inpatient settings, priority was often the medical acuity of the patient.

I know on the medical ward it's, ‘what's stopping them from going home’? Let's address that to get their goal to be able to go home (Physiotherapist; inpatient care).

Participants were cognisant of an institutional focus on impairments and associated discharge-based treatment goals. Participants needed to undertake large caseloads with a high volume of throughput. As a result, NTPA promotion was determined by the priority afforded to it by the clinician within the patient's episode of care.

When our prioritisation dictates that we need to see 15 people in a day, each person's only allocated enough time to get them to that level of discharge (Physiotherapist; inpatient care).

I think it comes down to a prioritisation thing. I think we see it as not as important as some of our other roles (Physiotherapist; outpatient care).

### 3.2 Clinicians believe that patient motivation influences NTPA promotion

Decisions to promote NTPA were influenced by low motivation on the part of patients. Many participants discussed that many patients have little to no interest in changing behaviour or becoming more physically active. When encountering patients with low motivation, some participants appeared to feel fatigued by this barrier. And in the absence of solutions to address this, they noted that it is easier to stick to the presenting condition, and forego NTPA promotion.

And then I guess there's the ones where I've tried a little bit or tried a few different tactics, and it's just gone nowhere. There doesn't seem to be any sort of interest in changing or anything like that … you go, ‘what's the point?’ (Physiotherapist; outpatient care).

Participants suggested that the frequency of NTPA promotion was likely influenced by negative experiences of dealing with unmotivated clients. Where previous attempts to promote NTPA with unmotivated patients have failed, clinician confidence in NTPA promotion was impacted and resulted in a narrowing of treatment to the presenting condition.

I think the reason we don't do it is we don't know how to do it effectively, like yes we're confident, we've got the knowledge and we know how to have that conversation … but what I think often the barrier comes up is the pushback from patients that might happen. And then you start losing your confidence or you may pre judge a patient … for example, if you get someone that says “what do you do for exercise?” “don't do anything”, “have you ever done exercise?” “don't do anything”, and the person [therapist] goes ‘oh well, what chance do I really have with this person’ and they just kind of ignore that and go straight into the rehab (AEP; outpatient care).

### 3.3 Clinicians rely on their professional and interpersonal skills and exposure to appropriate training to promote NTPA

When discussing NTPA promotion, the participants’ use of traditionally taught skills was prevalent. Goal setting was the predominant tool used by participants to facilitate NTPA promotion.

Goals, definitely. Really clear goals and setting multiple different goals as a bit of timeframes and things to achieve along the way (Physiotherapist; inpatient care).

Additional skills used included education provision and the use of activity diaries; skills used in general rehabilitation treatments. When these traditional skills were not effective in changing patient motivation or behaviour, many participants noted a lack of strategies to influence behaviour change.

And there are those [patients where] I'm not confident that I'm going to be able to get through to them. With some of them I might try for a couple of sessions and then I'll go, "Well, I don't think I'm ever going to get anything out of this person." Sort of give up, in a way. I've given them a fair bit of education, I've tried a few different strategies to try and motivate them, get them to do it, but doesn't seem like it's getting through. I just don't really feel like I have another option (Physiotherapist; outpatient care).

The three themes were mapped to four components of the COM-B model (Table 3). We identified four intervention functions that were relevant to facilitate NTPA promotion in routine hospital care: (1) enablement (increasing means/reducing barriers to increase capability); (2) environmental restructuring (changing the available resources); (3) training (imparting skills); and (4) education (increasing knowledge or understanding). Table 3 demonstrates the mapping of themes to intervention functions.

**Table 3:** Qualitative themes mapped to COM-B components and associated intervention functions.

Theme	COM-B Component	Intervention function	Example
1. Clinicians prioritise time to presenting condition	• Reflective Motivation	→ Education	Educate clinicians to build knowledge on behaviour change to increase capacity to integrate NTPA into routine care
	• Physical opportunity	→ Environmental restructuring; → Enablement	Inclusion of PA screening tools on assessment forms to facilitate NTPA discussions
2. Clinicians believe that patient motivation influences NTPA promotion	• Psychological capability	→ Training	Train clinicians to utilise behaviour change techniques and skills to influence patient motivation
3. Clinicians reliant on their professional and interpersonal skills and exposure to appropriate training to promote NTPA	• Social opportunity	→ Environmental restructuring; → Enablement	Provision of support meetings/community of practice to facilitate NTPA promotion within routine care

COM-B: Capability, Opportunity, Motivation, Behaviour; NTPA: Non-treatment physical activity; PA: Physical activity.

The quality of this study can be considered against the criteria proposed by Tracy et al., (2010) on the components of good qualitative investigation [20]. The alignment of this current study to the criteria and its effect on the rigor of the study is detailed in Supplement G.

## 4 Discussion

Physiotherapists and AEPs practicing in a regional public hospital setting in Australia consider NTPA promotion to be part of their role, report high levels of confidence and knowledge in promoting NTPA, and believe that it can help patients increase their PA away from the clinical setting. However, approaches to NTPA promotion are not systematic and are reliant on clinicians' decisions to promote NTPA, or not. Clinicians' promotion of NTPA is influenced by the priority they assign to it within the context of the patient's care journey, the patients' perceived motivation to increase PA, and the clinician's confidence in addressing behaviour change with unmotivated patients.

The reported frequency of 68% for NTPA promotion in our study broadly reflects the rates of NTPA promotion by physiotherapists and AEPs across published studies [[10], [11], [12]]. Initiatives such as ‘make every contact count’ advocate that clinicians use every patient interaction as a change to promote beneficial behaviours such as PA [21]. Given the high self-reported rates of confidence and knowledge in NTPA promotion, 34% of our respondents did not always promote NTPA. Crisford and colleagues found that 62% of Australian podiatrists promote NTPA, despite their respondents reporting lower knowledge and skills in NTPA promotion than observed in our study [22]. Given physiotherapists and AEPs are considered experts in PA [21] the reported practice suggests a missed opportunity to contribute to broader public health efforts and potentially reduce the burden of chronic diseases by promoting NTPA with all patients.

The frequency of NTPA promotion was heavily influenced by the priority afforded to it by the clinicians, and the fact that NTPA promotion does not follow a systematic approach within practice. This is a common challenge in healthcare settings, where clinicians focus on medical acuity, safety, and functional deficits [23]. Functional-restoration goals are often limited to those required for safe discharge, reinforcing the low priority assigned to more holistic NTPA promotion. Clinicians working in intensive care units or acute medical wards might see themselves as more closely aligned to the core aims of physiotherapy (restore movement and function when someone is affected by injury, illness or disability) than to the broader aims of health promotion [9]. These factors likely contributed to the lower rates of NTPA promotion observed in acute care, a finding supported by research examining hospital based practice [14]. Reductionist healthcare approaches decrease the scope for long-term care [5,14], and our qualitative interviews highlighted how patients often return for subsequent episodes of care with diminished PA capacity. Despite this, 68% of survey respondents never or rarely refer patients to community organisations and programs to support increasing PA. This finding highlights the need for greater emphasis on preventative health promotion practice and subsequent referral pathways into these dedicated community programs to support 10.13039/100006131PA promotion [24].

The motivation of patients influenced NTPA promotion by physiotherapists and AEPs in this study. Clinicians’ confidence in promoting NTPA decreased from 97% to 54% when working with unmotivated patients. The use of specific behaviour change techniques (BCTs) [25] was seldom identified in the interviews, although this does not suggest that they are not used. Identified strategies for motivating patients included education and goal setting, the latter being non-specific in nature and aligned to clinical documentation. For example, no distinction was made between goal setting (behaviour) and goal setting (outcome), which behaviour change science regards as distinct [25]. Clinicians must understand that for individuals unwilling or ambivalent towards change, the provision of information alone is often insufficient to achieve change and defined BCTs are required [26]. The lack of behaviour change skills has been reported to decrease NTPA promotion in physiotherapists working in outpatient musculoskeletal settings [8].

Only 54% of survey respondents in our study felt that the hospital provided the resources necessary to deliver NTPA promotion. Screening for PA levels strongly predicts PA promotion [12]. Other hospitals have integrated PA calculators into electronic medical records, ensuring that a baseline PA measure is taken at the initial consultation [27]. Providing healthcare practitioners with appropriate resources and PA promotion pathways facilitates discussions on PA and subsequent referrals to PA promotion programs [27]. As an example, surgeons practicing in ambulatory hospital settings markedly increased PA promotion by integrating a referral pathway to dedicated PA promotion program into routine care [28]. Therefore, from a practical perspective, having a systematic approach to preventive health is likely to increase PA promotion in the hospital setting.

Mapping of themes to the COM-B model highlighted that a multi-level strategic approach to creating behaviour change of the staff is required to support NTPA promotion [18,25]. Upskilling, empowering and equipping clinicians with techniques to change behaviour could support them to promote 10.13039/100006131PA, as well as other important health behaviours (e.g. diet, smoking). To facilitate ongoing 10.13039/100006131PA promotion by clinicians this education should not be delivered by one-off workshops, rather it requires an ongoing support and education program (e.g., role-playing, modelling, observing) over several months to facilitate maintenance of using any newly taught behaviour change strategies [29].

This study was the first mixed-methods investigation of NTPA promotion by physiotherapists and AEPs practicing in regional public hospital settings. Frequency of NTPA promotion has largely been measured using cross-sectional design [11,12,30]. The mixed-methods design employed in our study is a significant strength as it permitted an in-depth and rigorous exploration of the clinicians’ diverse approaches, experiences and perspectives. However, this study is not without limitations. First, NTPA practice was assessed via self-report. The accuracy of using self-report to assess NTPA promotion compared to other observational methods (e.g. filming of actual practice) is unclear, however this approach is consistent with numerous previous studies. Second, a degree of selection bias could have resulted from the high survey non-response rates and interview non-participation rates, with the participating clinicians potentially more engaged in NTPA promotion than non-responders. Third, 66% of survey respondents practiced in outpatient settings. The frequency of NTPA promotion was higher in outpatient settings compared to inpatient settings and this may have influenced overall reported frequency of NTPA promotion. The ratio of inpatient to outpatient clinicians practicing in the study hospital is close to that of the respondents, but does limit the generalisability of the findings to hospital settings with differing clinician ratios. Finally, this study was undertaken in a single hospital. Although single-site studies can limit generalisability, the primary aim of this research was to gain detailed knowledge about context and processes of NTPA promotion in this hospital to inform future health promotion planning. Steps were taken to maximise rigor (Supplement G) and this increases the broad applicability of the findings to other public hospital settings.

## 5 Conclusion

This study found many physiotherapists and AEPs promote NTPA with patients in an Australian regional hospital, with AEPs being greater promoters of NTPA. Physiotherapists and AEPs indicated that NTPA promotion is compatible with daily practice, although there is need for better guidance in how NTPA promotion is delivered. Strategies to increase NTPA promotion need to consider institutional and professional barriers, use the strengths of a supportive workplace and look to build pathways to integrate effective PA promotion into routine hospital care.

## 6 Practical implications

• NTPA is being promoted by physiotherapists and AEPs practicing in an Australian regional hospital, and clinicians believe NTPA promotion can help patients increase their general PA.
• Strategies such as integrating PA screening within clinical assessments may have the potential to increase frequency of NTPA promotion by physiotherapists and AEPs.
• The provision of education and training on NTPA promotion, and institutional guidelines and resources may reduce barriers to NTPA promotion in hospital setting.

## Confirmation of ethical compliance

Ethical approval was obtained from the Bendigo Health Care Group Ethics Committee and the La Trobe University Human Research Ethics Committee (reference number: LNR/72566/BH-2021-253248). All participants gave informed consent before data collection began.

## Funding information

Financial support was received from the Holsworth Research Initiative for the transcription of audio files. The funding source had no role in study design, data collection, data analysis, data interpretation or writing of the report.

## Author contributions

**CRediT author statement**: **Stephen Barrett:** Conceptualization, Methodology, Software, Investigation, Data curation, Formal analysis, Writing- Original draft preparation. **Kane Rodda:** Conceptualization, Methodology, Formal analysis, Writing- Reviewing and Editing. **Owen Howlett:** Methodology, Formal analysis, Writing- Reviewing and Editing. **Alistair Mumford:** Conceptualization, Methodology, Investigation, Formal analysis, Writing- Reviewing and Editing. **Donna Borkowski**: Conceptualization, Methodology, Formal analysis, Writing- Reviewing and Editing. **Josh Naunton:** Conceptualization, Methodology, Formal analysis, Writing- Reviewing and Editing. **Stephen Begg**: Conceptualization, Methodology, Formal analysis, Writing- Reviewing and Editing. **Breanne Kunstler:** Methodology, Formal analysis, Writing- Reviewing and Editing, Supervision. **Marcos De Noronha:** Methodology, Formal analysis, Writing- Reviewing and Editing. **Michael Kingsley:** Conceptualization, Methodology, Formal analysis, Funding acquisition, Writing- Reviewing and Editing.

## Declaration of competing interest

The authors declare that they have no known competing financial interests or personal relationships that could have appeared to influence the work reported in this paper..
