# Supplementary material for: The promotion of non-treatment physical activity in physiotherapy and exercise physiology practice in an Australian regional hospital: A mixed-methods study
Source: JSAMS Plus. 2023 Jan 16;2:100020. doi: 10.1016/j.jsampl.2023.100020 (PMC13008451; doi:10.1016/j.jsampl.2023.100020)
Supplement: Multimedia component 1 [file mmc1.docx]

Supplement A. Mixed-methods integration

| **Phase** | **Procedure** | **Product** |
| --- | --- | --- |
| Quantitative data  collection | Clinician survey | Self-reported frequency of NTPA and beliefs about preventive health |
|  |  |  |
| Quantitative data  analysis | Descriptive statistics | Building approach to integration through use of data analysis to inform interview guide |
|  |  |  |
| Interview participant sampling | Purposefully sample participants.  Develop interview questions and probes | Interview guide |
|  |  |  |
| Qualitative data  collection | Conduct individual interviews with clinicians | Interview recordings and transcripts |
|  |  |  |
| Qualitative data  analysis | Coding and thematic analysis | Themes and verbatim quotes |
|  |  |  |
| Integration of the  quantitative &  qualitative results | Interpretation and explanation of quantitative and qualitative results | Narrative integration in Discussion section using a contiguous mixed-methods approach |

NTPA: Non treatment physical activity
