# Supplementary material for: The promotion of non-treatment physical activity in physiotherapy and exercise physiology practice in an Australian regional hospital: A mixed-methods study
Source: JSAMS Plus. 2023 Jan 16;2:100020. doi: 10.1016/j.jsampl.2023.100020 (PMC13008451; doi:10.1016/j.jsampl.2023.100020)
Supplement: Multimedia component 2 [file mmc2.pdf]

Welcome.

This questionnaire will be used to identify the factors that influence your decision to promote non-treatment physical activity (NTPA) to patients.

Non-treatment physical activity is physical activity that is used to improve a patient's general health, as opposed to rehabilitating an ailment. Please keep this definition in mind as you progress through the questionnaire.

As an example, encouraging a person who is 1/52 post Total Knee Replacement to walk to their letterbox each day to get their knee moving is promoting physical activity for rehabilitation of an ailment.
If the individual was 6/52 post surgery, promoting 30 minutes of walking per day to enhance general health, and not specifically the knee is non-treatment physical activity promotion.

The questionnaire is expected to take approximately 15 - 20 minutes.

Please read the plain language statement before beginning the questionnaire.

Submission of the questionnaire is taken as consent to participate in this study.

Thank you very much for taking the time to complete our questionnaire. By doing so, you are making an important contribution to improving the ability of physiotherapists to help patients become physically active.

QuestionPro

* How old were you on your last birthday?

- ◯ Under 18
- ◯ 18 - 24
- ◯ 25 - 34
- ◯ 35 - 44
- ◯ 45 - 54
- ◯ 55 - 64
- ◯ 65 - 74
- ◯ 75 - 84
- ◯ 85 or older
- ◯ Prefer not to answer

* What is your gender?

- ◯ Male, or identifying as male
- ◯ Female, or identifying as female
- ◯ Prefer not to answer

* How many years have you been a practicing **physiotherapist** in Australia?

- 0-2
- 3-5
- 6-10
- 11-15
- 16+
- I am not registered to practice as a physiotherapist in Australia

* How many years have you been a practicing **accredited exercise physiologist** (AEP) in Australia?

- 0-2
- 3-5
- 6-10
- 11-15
- 16+
- I am not registered to practice as an AEP in Australia

QuestionPro

* Of these clinical settings, which do you work in **the most** in an average week?

- ○ Inpatient - acute
- ○ Inpatient - rehab
- ○ Outpatient - patients seen in clinic setting
- ○ Outpatient - patients seen in community setting (including home visits)
- ○ Other (please write)

Please enter here

* How many patients do you see 1:1 each week?

- ○ 0
- ○ 1-5
- ○ 6-15
- ○ 16-25
- ○ 26+

* How many patients do you see in group settings each week?

- ○ 0
- ○ 1-5
- ○ 6-15
- ○ 16-25
- ○ 26+

* As a reminder, **non-treatment physical activity** is physical activity that is used to improve a patient's general health, as opposed to rehabilitating an ailment.

Thinking about your clinical practice in the last month - specifically in the clinical area you practice in most, how often did you encourage your patients to have a more physically active lifestyle specifically for general health purposes?

- Never
- Rarely
- Sometimes
- Often
- All of the time

* Considering the patients you identified who would benefit from a NTPA intervention **in the past month,** what percentage of these patients did you **provide verbal NTPA advice** to:

- None
- 1 - 25%
- 26 - 50%
- 51 - 75%
- > 75%
- Did not identify any patients who would benefit from a NTPA intervention

* Considering the patients you identified who would benefit from a NTPA intervention **in the past month**, what percentage of these patients did you **provide written NTPA advice (e.g. pamphlet, summary sheet)** to:

- None
- 1 - 25%
- 26 - 50%
- 51 - 75%
- > 75%
- Did not identify any patients who would benefit from a NTPA intervention

* Considering the patients you identified who would benefit from a NTPA intervention **in the past month**, what percentage of these patients did you **refer to other service providers/agencies for NTPA support (e.g. strength training groups, walking groups)**:

- None
- 1 - 25%
- 26 - 50%
- 51 - 75%
- > 75%
- Did not identify any patients who would benefit from a NTPA intervention

* (Aside from your physiotherapy or AEP degree), do you have any qualifications in psychology or have you attended at least 10 hours of educational classes with a psychology focus? Topics might include motivational interviewing, health coaching, cognitive behavioural therapy or pain management.

- Yes
- No
- Unsure

Please specify what additional qualification/training you have completed

* (Aside from your physiotherapy or AEP degree), do you have any qualifications in health promotion or have you attended at least 10 hours of educational classes with a health promotion focus? Topics might include physical activity promotion, smoking cessation, healthy eating, weight loss and alcohol consumption.

- Yes
- No
- Unsure

Please specify what additional qualification/training you have completed

* In the past week, on **how many days** have you done a total of 30 min or more of **moderate-to-vigorous physical activity**, which was enough to raise your breathing rate?

This may include sport, exercise and brisk walking or cycling for recreation or to get to and from places, but should not include housework or physical activity that may be part of your job.

- ○ 0
- ○ 1
- ○ 2
- ○ 3
- ○ 4
- ○ 5
- ○ 6
- ○ 7

* In the past week, on **how many days** have you participated in **muscle strengthening** activities?

Muscle strengthening activities are activities for improving strength, power, endurance and size of skeletal muscles.

This may include body weight exercises, free weights, resistance machines, and pilates.

- ◯ 0
- ◯ 1
- ◯ 2
- ◯ 3
- ◯ 4
- ◯ 5
- ◯ 6
- ◯ 7

Note: When answering the following questions, please answer in relation to the clinical setting in which you **work in the most** in an average week.

If you hold a managerial role, some of these questions might not apply to you. In this instance, please choose 'Not applicable'

QuestionPro

* As a reminder, **non-treatment physical activity** is physical activity that is used to improve a patient's general health, as opposed to rehabilitating an ailment.

When I consider using a non-treatment physical activity intervention in my practice:

	Strongly disagree	Somewhat disagree	Neither agree nor disagree	Somewhat agree	Strongly agree
I know how to deliver this intervention.	○	○	○	○	○
Objectives of this intervention and my role in this are clearly defined for me.	○	○	○	○	○
I DO NOT know what my responsibilities are.	○	○	○	○	○
I know exactly what is expected from me.	○	○	○	○	○
Delivering this intervention is part of my work as a physiotherapist or AEP.	○	○	○	○	○
As a physiotherapist or AEP, it is NOT my job to deliver this intervention.	○	○	○	○	○
It is my responsibility as a physiotherapist or AEP to deliver this intervention.	○	○	○	○	○
For me, delivering the content of the intervention is easy.	○	○	○	○	○
For me, performing the initial assessment of physical activity [illegible]	○	○	○	○	○

levels is easy.					
For me, giving attention to my patients' maintenance of physical activity behaviour outside the clinic is easy.	○	○	○	○	○

	Strongly disagree	Somewhat disagree	Neither agree nor disagree	Somewhat agree	Strongly agree
I intend to deliver this intervention in the next three months.	○	○	○	○	○
My intention to deliver this intervention in the next three months is strong.	○	○	○	○	○
I will definitely deliver this intervention in the next three months.	○	○	○	○	○
It is possible for me to tailor this intervention to my patients' needs.	○	○	○	○	○
This intervention takes little time to deliver.	○	○	○	○	○
This intervention is well-suited to daily practice.	○	○	○	○	○
This intervention is simple to deliver.	○	○	○	○	○
Most people who are important to me in a professional sense think that I should deliver this intervention.	○	○	○	○	○
I have been trained in delivering this intervention.	○	○	○	○	○

	Strongly disagree	Somewhat disagree	Neither agree nor disagree	Somewhat agree	Strongly agree
I DO NOT have the skills to deliver this intervention.	◯	◯	◯	◯	◯
I have experience delivering this intervention.	◯	◯	◯	◯	◯

QuestionPro

* As a reminder, **non-treatment physical activity** is physical activity that is used to improve a patient's general health, as opposed to rehabilitating an ailment.

When I consider using a non-treatment physical activity intervention in my practice:

	Strongly disagree	Somewhat disagree	Neither agree nor disagreee	Somewhat agree	Strongly agree	Not applicable
I can count on support from the management of my workplace when things get tough.	◯	◯	◯	◯	◯	◯
The management of my workplace is willing to listen to my problems with delivering this intervention.	◯	◯	◯	◯	◯	◯
The management of my workplace is helpful when delivering this intervention.	◯	◯	◯	◯	◯	◯
Professionals with whom I work are willing to listen to my problems with delivering this intervention.	◯	◯	◯	◯	◯	◯
Professionals with whom I work are helpful with delivering this intervention.	◯	◯	◯	◯	◯	◯

* As a reminder, non-treatment physical activity is physical activity that is used to improve a patient's general health, as opposed to rehabilitating an ailment.

When I consider using a non-treatment physical activity intervention in my practice, **my workplace provides**:

	Strongly disagree	Somewhat disagree	Neither agree nor disagree	Somewhat agree	Strongly agree	Not applicable
All necessary resources to deliver this intervention.	◯	◯	◯	◯	◯	◯
Training to deliver this intervention.	◯	◯	◯	◯	◯	◯
Sufficient intervention materials to support implementation and delivery.	◯	◯	◯	◯	◯	◯
Assistance with delivering this intervention.	◯	◯	◯	◯	◯	◯
Support meetings where I can get my questions answered about delivering this intervention.	◯	◯	◯	◯	◯	◯

* When I consider using a non-treatment physical activity intervention in my practice, **I am confident** that I can:

	Strongly disagree	Somewhat disagree	Neither agree nor disagree	Somewhat agree	Strongly agree
Deliver this intervention.	◯	◯	◯	◯	◯
Deliver this intervention even when there is little time.	◯	◯	◯	◯	◯
Deliver this intervention even when patients are not motivated.	◯	◯	◯	◯	◯

* When I consider using a non-treatment physical activity intervention in my practice, addressing other patient problems:

	Strongly disagree	Somewhat disagree	Neither agree nor disagree	Somewhat agree	Strongly agree
Are a higher priority than delivering this intervention.	◯	◯	◯	◯	◯
Are more urgent than delivering this intervention.	◯	◯	◯	◯	◯

* Patients receiving non-treatment physical activity interventions from me:

	Strongly disagree	Somewhat disagree	Neither agree nor disagree	Somewhat agree	Strongly agree
Are motivated to do it.	◯	◯	◯	◯	◯
Are NOT positive about the intervention.	◯	◯	◯	◯	◯

* When considering non-treatment physical activity interventions, I have a clear plan:

	Strongly disagree	Somewhat disagree	Neither agree nor disagree	Somewhat agree	Strongly agree
How I will deliver this intervention.	○	○	○	○	○
Under what circumstances I will deliver this intervention.	○	○	○	○	○
How to deliver this intervention when patients are not motivated.	○	○	○	○	○
How to deliver this intervention when there is little time.	○	○	○	○	○

* In my general work as a physiotherapist or AEP:

	Strongly disagree	Somewhat disagree	Neither agree nor disagree	Somewhat agree	Strongly agree
In uncertain times, I usually expect the best.	○	○	○	○	○
I am never optimistic about the future.	○	○	○	○	○
Overall, I expect more good things to happen than bad.	○	○	○	○	○

* If I deliver a non-treatment physical activity intervention:

	Strongly disagree	Somewhat disagree	Neither agree nor disagree	Somewhat agree	Strongly agree
It will be effective.	◯	◯	◯	◯	◯
My patients will be appreciative.	◯	◯	◯	◯	◯
It will NOT help patients become more physically active.	◯	◯	◯	◯	◯

* For me, delivering a non-treatment physical activity intervention:

	Strongly disagree	Somewhat disagree	Neither agree nor disagree	Somewhat agree	Strongly agree
Is worthwhile.	◯	◯	◯	◯	◯

* When I deliver a non-treatment physical activity intervention, I feel:

	Strongly disagree	Somewhat disagree	Neither agree nor disagree	Somewhat agree	Strongly agree
Optimistic	◯	◯	◯	◯	◯
Nervous	◯	◯	◯	◯	◯
Cheerful	◯	◯	◯	◯	◯
Comfortable	◯	◯	◯	◯	◯
Pessimistic	◯	◯	◯	◯	◯
Uncomfortable	◯	◯	◯	◯	◯

* When I deliver a non-treatment physical activity intervention:

	Strongly disagree	Somewhat disagree	Neither agree nor disagree	Somewhat agree	Strongly agree
I get recognition from the management at work.	○	○	○	○	○
I get recognition from my peers at work.	○	○	○	○	○

* Delivering a non-treatment physical activity intervention is something:

	Strongly disagree	Somewhat disagree	Neither agree nor disagree	Somewhat agree	Strongly agree
I do automatically.	○	○	○	○	○
I do without having to consciously remember.	○	○	○	○	○
I do without thinking.	○	○	○	○	○
I often forget.	○	○	○	○	○

Do you have anything else to add?

Please feel free to provide any comments or additional information you would like to share on the topic of promotion of non-treatment physical activity in the hospital setting.

Interested in participating in the next stage of this research?

The second part of this research project involves carrying out semi-structured interviews with a number of physiotherapists and AEPs. The findings from the surveys will be explored with a number of physiotherapists and AEPs to assist with informing education and clinical practice.

To do this, we need people to volunteer their interest in participating in a semi-structured interview. If you are interested in participating in a semi-structured interview please enter your name and email address below.

Please note that there is a possibility that not all individuals who express an interest in participating in a semi-structured interview will be required.

If you do not want to participate in the interviews study, simply press 'Next'

Name

Email address

La Trobe University
