# Supplementary material for: The promotion of non-treatment physical activity in physiotherapy and exercise physiology practice in an Australian regional hospital: A mixed-methods study
Source: JSAMS Plus. 2023 Jan 16;2:100020. doi: 10.1016/j.jsampl.2023.100020 (PMC13008451; doi:10.1016/j.jsampl.2023.100020)
Supplement: Multimedia component 3 [file mmc3.docx]

Supplement C. Interview guide

*Provide the interviewee with the definition of NTPA*

Provide interviewee opportunity to clarify the definition.

To start, can you tell me a little bit about your main area of work, and what a typical patient presentation may be?

How many times would you see a patient on average?

What are your professional priorities with these patients?

Q. There was a study done in 2018 that showed that 43% of PTs ALWAYS promoted NTPA to patients.

Our sample size is smaller, and the results showed that 32% of PT/AEPs ALWAYS promoted NTPA to patients.

Do you have any thoughts about these findings?

- Are those numbers high/low
- Are they what you would expect – why?

The 2018 study was mostly on clinicians in private practice, whereas our study was on hospital clinicians – do you think working in PP or public makes a difference to NTPA practice?

Q. Now you understand the definition of NTPA, how compatible do you feel it is with your current practice?

*Prompts*

1. Do you see any benefit to promoting NTPA?

2. Can you tell me, do you think promoting NTPA is compatible with the role of physiotherapists/AEPs in general?

- Why/why not?

Q. Is there another profession that should have more responsibility than physiotherapists/AEPs for promoting NTPA?

- E.g.: psychologists, general practitioners and personal trainers.

Q. Do you refer patients to other professionals if they need help becoming physically active?

Q. Do you refer patients to other services if they need help becoming physically active?
 - e.g.: walking groups, strength training programs

**Topic: Training/Skills/Knowledge**

Q. “What kind of skills do you feel are beneficial in promoting NTPA to patients?

- Can you please tell me if you feel you have the skills to promote NTPA (and why?)”

*Prompts*

- Have you received specific training in NTPA promotion? If so, what was it?

Q. Can you please rate from 1 to 10 how confident you feel promoting NTPA to your patients?

- Can you please explain why you chose that score?
- *Prompt* - Why are you an 8 rather than a 6

Q. Have you learnt about NTPA at university?

- What about at professional development seminars or courses?

- Have you practiced promoting NTPA in any of these environments?

- Have you done anything to try and maintain or improve your skills promoting NTPA?

Q. Data from our group and other research indicates that PTs and EPs have high confidence and knowledge in promoting NTPA. The data also indicates that NTPA is within their professional capabilities.

Yet the rates of NTPA promotion are relatively low, around the 40% mark – do you have any thoughts on why clinicians who are confident and knowledgeable in a skill do not use it all the time?

**Topic: Barriers**

Q. Do you screen the patients/clients you see for their physical activity levels?

- If so, using what methods?

Q. If your patient has low activity levels, how would you prioritise the need to increase activity levels?

Q. Do you experience any barriers to promoting physical activity with the patients/clients you see?

*Prompts*

*- If so, what are they?*

Q. How much time would it take you to promote NTPA to a patient?

- What do you think about this?

- Is this amount of time too much or too little?

- Priorities?

**Topic: Planning**

Q. “Let’s talk about the patients/clients. What do you think patients/clients think about getting NTPA advice from clinicians?

Q. Have you tried to promote NTPA to a patient with low motivation?”

*Prompts*

1. How did you do this?

2. Do you think it worked?

3. Did you use any particular techniques?

4. Was it easy or hard? Why?

**Topic: Patient presentation**

Q. I would like you to consider if your decision to promote NTPA depends on patient presentation. Can you please tell me if there are any patient-specific things that would make you more or less likely to promote NTPA?”

*Prompts*

- Gender/weight/age/attitude of patient

- Would your decision to (or not to) promote NTPA change if these patients were undergoing treatment for breast cancer?

- Would NTPA promotion become a higher/lower/same priority given this information?

- Does this mean you would be more/less/same inclined to promote NTPA to this patient?

- What if, instead of being treated for cancer, this patient had a heart condition that was being managed by their doctor?

- Would NTPA promotion become a higher/lower/same priority given this information?

- Does this mean you would be more/less/same inclined to promote NTPA to this patient?

**Part 2: Clinicians’ use of BCTs when promoting NTPA?**

Q. Do you think it is within the physiotherapist/AEP scope of practice to change behaviour?

*Prompts*

1. If yes, why?

2. If no, why not?

*Provide interviewee with the definition of BCTs*

**Definition: A behavior change technique (BCT) is a systematic procedure included as an active component of an intervention designed to change behavior.**

*Provide interviewee opportunity to clarify the definition.*

Q. Considering behaviour change, what strategies do you use to change behaviour in general – needn’t be specific to physical activity

Q. Does your intention to change patient behaviour depend on anything?

a. E.g.: Evidence for efficacy, familiarity, social comparison, subjective norm, knowledge, skill, confidence, time, patient education level, patient presentation, treatment being delivered etc.

- What makes it easy to change behaviour?

- What makes it hard?

Q. What type of support would you find useful to help you to change patient behaviour?

*Prompts*

- Who should provide this support?

- What would this support look like?

- Is it important that physiotherapy/AEP students learn how to use behaviour change techniques?

- Is it important that practicing physiotherapists/AEPs learn how to use behaviour change techniques?

- Have you learnt how to use BCTs at university or during any professional development sessions?
