# Supplementary material for: The promotion of non-treatment physical activity in physiotherapy and exercise physiology practice in an Australian regional hospital: A mixed-methods study
Source: JSAMS Plus. 2023 Jan 16;2:100020. doi: 10.1016/j.jsampl.2023.100020 (PMC13008451; doi:10.1016/j.jsampl.2023.100020)
Supplement: Multimedia component 4 [file mmc4.docx]

Supplement D. Participant responses of statements on behaviours, beliefs and attitudes regarding NTPA promotion

| **Domain** | **Items** | **Scale** | **Mean**  **(SD)** | **Number of responses (%)** | **Number of responses (%)** | **Number of responses (%)** |
| --- | --- | --- | --- | --- | --- | --- |
|  |  |  |  | *Agree or strongly agree* | *Neither disagree nor agree* | *Strongly disagree or Disagree* |
| **Behavioural regulation** | I have a clear plan under what circumstances I will deliver this intervention (n = 35) | 1 - 5 | 3.8 (0.89) | **28 (80.0)** | 4 (11.4) | 3 (8.6) |
|  | I have a clear plan of how I will deliver this intervention (n = 35) | 1 - 5 | 3.7 (0.9) | **27 (77.2)** | 4 (11.4) | 4 (11.4) |
|  | I have a clear plan of how to deliver this intervention when patients are not motivated (n = 35) | 1 - 5 | 3.2 (1.1) | **16 (45.7)** | 10 (28.6) | 9 (25.7) |
|  | I have a clear plan of how to deliver this intervention when there is little time (n = 35) | 1 - 5 | 3.3 (1.1) | **17 (48.6)** | 8 (22.9) | 10 (28.6) |
| **Beliefs about capabilities** | For me, performing the initial assessment of physical activity levels is easy (n = 39) | 1 - 5 | 3.9 (1.0) | **29 (74.3)** | 6 (15.4) | 4 (10.3) |
|  | For me, delivering the content of the intervention is easy (n = 39) | 1 - 5 | 3.9 (0.8) | **29 (74.3)** | 9 (23.1) | 1 (2.6) |
|  | For me, giving attention to my patients’ maintenance of physical activity behaviour outside the clinic is easy (n = 35) | 1 - 5 | 3.3 (1.1) | **19 (54.3)** | 8 (22.9) | 8 (22.9) |
|  | I am confident that I can deliver this intervention  (n = 35) | 1 - 5 | 3.3 (1.1) | **34 (97.1)** | 0 (0) | 1 (2.9) |
|  | I am confident that I can deliver this intervention even when there is little time (n = 35) | 1 - 5 | 3.3 (1.1) | **25 (71.4)** | 6 (17.1) | 4 (11.4) |
|  | I am confident that I can deliver this intervention even when patients are not motivated (n = 35) | 1 - 5 | 3.3 (1.1) | **19 (54.3)** | 9 (25.7) | 7 (20.0) |
| **Goals** | Addressing other patient problems are a higher priority than delivering this intervention (n = 35) | 1 - 5 | 3.6 (1.0) | **21 (60.0)** | 9 (25.7) | 5 (14.3) |
|  | Addressing other patient problems are more urgent than delivering this intervention (n = 35) | 1 - 5 | 3.6 (1.0) | **22 (62.9)** | 8 (22.9) | 5 (14.3) |
| **Beliefs about consequences** | My patients will be appreciative if I deliver a non-treatment physical activity intervention (n = 35) | 1 - 5 | 3.6 (0.8) | **23 (65.7)** | 10 (28.6) | 2 (5.7) |
|  | If I deliver a non-treatment physical activity intervention, it will not help my patients will become more physically active (n = 35) | 1 - 5 | 4.6 (1.0) | **32 (91.4)** | 3 (8.6) | 0 (-) |
|  | If I deliver a non-treatment physical activity intervention, it will be effective (n = 35) | 1 - 5 | 3.8 (0.8) | **26 (74.3)** | 7 (20.0) | 2 (5.7) |
|  | For me, delivering a non-treatment physical activity intervention is worthwhile (n = 35) | 1 - 5 | 4.5 (0.8) | **34 (97.1)** | 1 (2.9) | 0 (-) |
|  | I get recognition from my peers at work when I deliver a non-treatment physical activity intervention (n = 35) | 1 - 5 | 2.4 (0.9) | 3 (8.6) | 12 (34.3) | **20 (57.1)** |
| **Innovation** | It is possible for me to tailor this intervention to my patients’ needs (n = 39) | 1 - 5 | 4.4 (0.78) | **37 (94.8)** | 1 (2.6) | 1 (2.6) |
|  | This intervention takes little time to deliver (n = 39) | 1 - 5 | 3.4 (1.1) | **20 (51.3)** | 9 (23.1) | 10 (25.6) |
|  | This intervention is well suited to daily practice (n = 39) | 1 - 5 | 4.0 (1.2) | **32 (82.1)** | 1 (2.6) | 6 (15.3) |
|  | This intervention is simple to deliver (n = 39) | 1 - 5 | 3.9 (0.9) | **32 (82.1)** | 4 (10.3) | 3 (7.6) |
| **Innovation strategy** | My workplace provides training to deliver this intervention (n = 35) | 1 - 5 | 2.8 (1.1) | 11  (31.4) | 7 (20.0) | **17 (48.6)** |
|  | My workplace provides sufficient intervention materials to support implementation and delivery (n = 35) | 1 - 5 | 3.1 (1.1) | **15 (42.9)** | 6 (17.1) | 14 (40.0) |
|  | My workplace provides assistance with delivering this intervention (n = 35) | 1 - 5 | 3.2 (1.0) | **13 (37.1)** | **13 (37.1)** | 9 (25.8) |
|  | My workplace provides support meetings where I can get my questions answered about delivering this intervention (n = 35) | 1 - 5 | 2.9 (1.2) | 11 (31.4) | 8 (22.9) | **16 (45.7)** |
| **Intentions** | I intend to deliver this intervention in the next three months (n = 39) | 1 - 5 | 4.5 (0.9) | **35 (89.7)** | 3 (7.6) | 1 (2.6) |
|  | My intention to deliver this intervention in the next three months is strong (n = 39) | 1 - 5 | 4.4 (1.0) | **30 (85.7)** | 4 (10.2) | 1 (2.9) |
|  | I will definitely deliver this intervention in the next three months (n = 39) | 1 - 5 | 4.3 (1.1) | **31 (79.4)** | 6 (15.4) | 2 (5.1) |
| **Knowledge** | I know how to deliver this intervention (n = 39) | 1 - 5 | 4.2 (0.8) | **35 (89.7)** | 3 (7.7) | 1 (2.6) |
|  | The objectives of this intervention and my role in this are clearly defined for me (n = 39) | 1 - 5 | 3.8 (0.9) | **28 (71.8)** | 7 (18.0) | 4 (10.2) |
|  | With regard to promoting non-treatment physical activity, I know what my responsibilities are (n = 39) | 1 - 5 | 4.2 (0.9) | **32 (82.1)** | 5 (12.8) | 2 (5.1) |
|  | With regard to promoting non-treatment physical activity, I know exactly what is expected from me (n = 39) | 1 - 5 | 3.7 (1.0) | **24 (61.6)** | 11 (28.2) | 4 (10.2) |
| **Nature of the behaviour** | Delivering a non-treatment physical activity intervention is something I do automatically (n = 35) | 1 - 5 | 4.1 (0.9) | **32 (91.4)** | 0 | 3 (8.6) |
|  | Delivering a non-treatment physical activity intervention is something I do without having to consciously remember (n = 35) | 1 - 5 | 3.9 (1.0) | **29 (82.8)** | 2 (5.7) | 4 (11.4) |
|  | Delivering a non-treatment physical activity intervention is something I do without thinking (n = 35) | 1 - 5 | 3.8 (1.1) | **28 (80.0)** | 2 (5.7) | 5 (14.3) |
|  | Delivering a non-treatment physical activity intervention is something I often forget to do (n = 35) | 1 - 5 | 2.0 (0.9) | 3 (8.6) | 5 (14.3) | **27 (77.2)** |
| **Patient** | Patients receiving non-treatment physical activity interventions from me are motivated to do it (n = 35) | 1 - 5 | 3.2 (0.8) | 13 (37.1) | **16 (45.7)** | 6 (17.1) |
|  | Patients receiving non-treatment physical activity interventions from me are positive about the intervention (n = 35) | 1 - 5 | 3.4 (0.8) | **16 (45.7)** | 13 (37.1) | 6 (17.1) |
| **Negative emotions** | I feel nervous when I deliver a non-treatment physical activity intervention (n = 35) | 1 - 5 | 1.7 (0.8) | 0 | 6 (17.1) | **29 (82.8)** |
|  | I feel pessimistic when I deliver a non-treatment physical activity intervention (n = 35) | 1 - 5 | 1.8 (0.9) | 3 (8.6) | 2 (5.7) | **30 (85.7)** |
|  | I feel uncomfortable when I deliver a non-treatment physical activity intervention (n = 35) | 1 - 5 | 1.5 (0.7) | 0 | 4 (11.4) | **31 (88.5)** |
| **Optimism** | In my work as a PT/AEP, in uncertain times, I usually expect the best (n = 35) | 1 - 5 | 3.6 (1.0) | **22 (62.9)** | 9 (25.7) | 4 (11.4) |
|  | In my work as a PT/AEP I am never optimistic about the future (n = 35) | 1 - 5 | 1.4 (1.0) | 0 | 0 | **35 (100)** |
|  | In my work as a PT/AEP, overall, I expect more good things to happen than bad (n = 35) | 1 - 5 | 4.2 (0.9) | **31 (88.5)** | 2 (5.7) | 2 (5.7) |
| **Organisation** | My workplace provides all necessary resources to deliver this intervention (n = 35) | 1 - 5 | 3.3 (1.2) | **19 (54.3)** | 4 (11.4) | 12 (34.3) |
|  | The management of my workplace is willing to listen to my problems with delivering this intervention (n = 36) | 1 - 5 | 4.1 (1.0) | **27 (75.0)** | 7 (19.4) | 2 (5.6) |
|  | The management of my workplace is helpful when delivering this intervention (n = 35) | 1 - 5 | 4.1 (1.2) | **25 (71.4)** | 6 (17.1) | 4 (11.4) |
|  | I can count on support from the management of my workplace when things get tough around promoting non-treatment physical activity (n = 36) | 1 - 5 | 4.1 (1.1) | **28 (77.7)** | 5 (13.8) | 3 (7.8) |
| **Organisation** | My workplace provides all necessary resources to deliver this intervention (n = 35) | 1 - 5 | 3.3 (1.2) | **19 (54.3)** | 4 (11.4) | 12 (34.3) |
| **Positive emotions** | I feel optimistic when I deliver a non-treatment physical activity intervention (n = 35) | 1 - 5 | 4.0 (0.8) | **31 (88.5)** | 3 (8.6) | 1 (2.9) |
|  | I feel cheerful when I deliver a non-treatment physical activity intervention (n = 35) | 1 - 5 | 3.9 (0.8) | **28 (80.0)** | 6 (17.1) | 1 (2.9) |
|  | I feel comfortable when I deliver a non-treatment physical activity intervention (n = 35) | 1 - 5 | 4.2 (0.8) | **32 (91.4)** | 2 (5.7) | 1 (2.9) |
|  |  |  |  |  |  |  |
| **Skills** | I have been trained in delivering this intervention (n = 39) | 1 - 5 | 3.6 (1.1) | **22 (61.5)** | 8 (23.1) | 6 (15.4) |
|  | I do not have the skills to deliver this intervention (n = 39) | 1 - 5 | 1.6 (0.7) | 1 (2.6) | 2 (5.1) | **36 (92.3)** |
|  | I have experience delivering this intervention (n = 39) | 1 - 5 | 4.2 (0.9) | **34 (87.2)** | 2 (5.1) | 3 (7.7) |
|  |  |  |  |  |  |  |
| **Social influences** | Most people who are important to me in a professional sense think that I should deliver this intervention (n = 39) | 1 - 5 | 4.0 (0.9) | **27 (69.2)** | 9 (23.1) | 1 (2.6) |
|  | Professionals with whom I work are willing to listen to my problems with delivering this intervention (n = 35) | 1 - 5 | 4.4 (1.0) | **31 (82.8)** | 4 (11.4) | 2 (5.7) |
|  | Professionals with whom I work are helpful with delivering this intervention (n = 35) | 1 - 5 | 3.6 (1.0) | **29 (88.6)** | 2 (5.7) | 2 (5.7) |
|  |  |  |  |  |  |  |
| **Social/professional role and identity** | Delivering this intervention is part of my work as a physiotherapist/AEP (n = 39) | 1 - 5 | 4.4 (0.9) | **36 (92.3)** | 0 | 3 (7.7) |
|  | As a PT/AEP, it is not my job to deliver this intervention (n = 39) | 1 - 5 | 1.3 (0.6) | 0 | 2 (5.1) | **37 (94.9)** |
|  | It is my responsibility as a PT/AEP to deliver this intervention (n = 39) | 1 - 5 | 4.4 (0.9) | **35 (89.7)** | 3 (7.7) | 1 (2.6) |

AEP: Accredited exercise physiologist; NTPA: Non-treatment physical activity; PT: Physiotherapist
