# Supplementary material for: The promotion of non-treatment physical activity in physiotherapy and exercise physiology practice in an Australian regional hospital: A mixed-methods study
Source: JSAMS Plus. 2023 Jan 16;2:100020. doi: 10.1016/j.jsampl.2023.100020 (PMC13008451; doi:10.1016/j.jsampl.2023.100020)
Supplement: Multimedia component 5 [file mmc5.docx]

Supplement E. Codes, categories and themes relating to physiotherapists and AEPs attitudes and beliefs towards NTPA promotion in hospital practice

| **Codes** | **Categories** | **Themes** |
| --- | --- | --- |
| - Look beyond the referral, consider the whole person and their needs. - Unsure if AEPs/PTs see NTPA as their responsibility. - Failure to recognise NTPA as part of role - Importance of discussing ongoing PA at multiple time points. - NTPA should be viewed as value adding. - Lacking knowledge and skills to address comorbidities - Experience level influences mastery and awareness of NTPA. - Self-driven decision to address ongoing exercise - Too early to refer to health promoting groups for some patients - Fixing the problem that is in front of us - Focus of therapy service - Requires time and regular contact to complete effective NTPA - Organisational expectation only to address rehab goals. - Consider whether patients really want to discuss PA - Easier to discuss PA relating to treating condition | - Hospital multi-discipline treatments and goals are set around presenting condition - Easier to engage in patients on presenting condition - Patient goals are set around presenting condition - Decision to promote NTPA is driven by individual clinician – not measured - No system that encourages NTPA | Clinicians prioritise addressing the presenting condition before NTPA |
| - Difficulty talking to people who are not physically active like you - Patient motivation is a barrier - Easier to stick to condition when you see that motivation is low - Takes too much time - Can tell that some people won't change - Other priorities for patients - Barriers to prescribing NTPA - time and environmental factors. - Failing to see the value add in promoting PA - Difficult to change people’s motivation - Clinical experience important for skill development in patient communication - Belief that some people just won’t change their PA - Increasing general PA is not one of the patients goals - Patient's characteristic and knowledge | - When perceived motivation is low it is easier to avoid NTPA and stick to presenting condition - Unconscious biases around PA - Concern that promoting NTPA to patients with low motivation takes too much time - patients often don’t set increasing PA as their goal | Clinicians believe that patient motivation influences NTPA promotion |
| - Learning about NTPA did not occur at university - Goal setting is the primary technique used - Further training further help me learn about NTPA - Motivational interviewing touched on at university, but not followed up - Unsure about specifics on behaviour change techniques - Being a good communicator can help - Graded exposure to exercise - Being confident can facilitate change - Empathy - Really try to educate the patient about activity - Provide support to encourage and grade the level of activity | - Uncertainty on BCT specifics/ taxonomy - Lack of details on specific MI skills - Acknowledge need to upskill in NTPA promotion - Use of traditional taught skills prevalent - Use of behaviour change skills less common - Progressive increases/loading in PA is different to motivating to engage in PA | Clinicians rely on their professional and interpersonal skills and exposure to appropriate training to promote NTPA |

AEP: Accredited exercise physiologist; NTPA: Non-treatment physical activity; PA: Physical activity; PT: Physiotherapist
