# Supplementary material for: The promotion of non-treatment physical activity in physiotherapy and exercise physiology practice in an Australian regional hospital: A mixed-methods study
Source: JSAMS Plus. 2023 Jan 16;2:100020. doi: 10.1016/j.jsampl.2023.100020 (PMC13008451; doi:10.1016/j.jsampl.2023.100020)
Supplement: Multimedia component 6 [file mmc6.docx]

Supplement F. Themes and verbatim quotes

| **Theme** | **Quote** |
| --- | --- |
| Clinicians prioritise addressing the presenting condition before NTPA | - When our prioritisation dictates that we need to see 15 people in a day, each person's only allocated enough time to get them to that level of discharge (Physiotherapist; inpatient care) - It's [NTPA] not a priority at the time. We tend to see people, and a big part of I guess our treatment is being very much goal based and focusing on people achieving their goals and then once they've achieved their goals, all right, can we discharge them? Can we get rid of them? It's sort of like generally you're quite focused on them achieving their goals, which tends to be condition specific, treatment specific (Physiotherapist; inpatient care) - The demands of the public system prioritising a health promotion message that may well benefit this person in the long run, but I can't show an immediate reason to see them, that's going to be priority two or three as opposed to a priority one, because someone's got a broken ankle or something that I'm seeing them for (Physiotherapist; outpatient care) - Get to a point where they're safe to be at home, but then can we get them to a point where they can have at least some of that self-management with some outpatient follow-up, as well (Physiotherapist; inpatient care) - The presentation is forever evolving and dynamic and things take priority very quickly and change very quickly. So when you think that might be time to approach that, and something else has happened, I think that happens a lot (AEP; outpatient care) - Basically, every area of the hospital, because it's quite goal based, I'd probably say that non-treatment physical activity's where you're promoting it in a non-goal based section of their ... Not so much of their therapy. Where it's just sort of promoting them just for the betterment of themselves in general and their general health. That's because there's such an emphasis on being goal setting, where once you've achieved those goals, the priority of that other stuff isn't as big (Physiotherapist; outpatient care) - I think it comes down to a prioritization thing. I think we see it as not as important as some of our other role (Physiotherapist; outpatient care) - Yeah so I guess our priorities would be addressing what they came in for referral (AEP; outpatient care) - I don't know if a lot of AEP'S or physios see it as their responsibility. I feel like a lot of physios or AEP's mostly is the rehab as their responsibility. That's where it ends (AEP; outpatient care) |
| Clinicians believe that patient motivation influences NTPA promotion | - I feel like a lot of them would... put it in the same category is when a doctor tells them to stop smoking, "I know, I know, I know it's good for my health". But I think a lot, kind of have that perspective. Everybody knows it's good for you to an extent and I think it comes across a bit, as you know, that what's the word... harping on advice. They just keep hearing it? "I know it's good for me. I know I should be doing it" (Physiotherapist; inpatient care) - Some are quite welcoming it and receive it well, at a point where they're ready to change and are welcoming of the information and input. Whereas some are probably not so ready to change, stuck in their ways, a bit lower motivation, that kind of thing (AEP; outpatient care) - I think another perspective is "I have enough problems as it is." So, I've got a lot of stuff going on already and most, in all fairness, a lot of them do. So it's not a priority (Physiotherapist; inpatient care) - It's probably based primarily on the fact that I'd probably say 50-60% of my clients I feel if I was promoting it to them, they would listen and they would take it on board and try and make those improvements. But then there's that other 40% where it's just their own motivation, or whether it's from cognitive issues, or just their lifestyle, whether they're too busy with other parts of their life, it's not a priority for them. It's those ones where it's really hard to get through to them, those 40% where I feel like I just really struggle to do that. Really struggle to connect and give them I guess that motivation to continue to do it and get them to feel like it's worth doing (Physiotherapist; inpatient care) - And then I guess there's the ones where I've tried a little bit or tried a few different tactics, and it's just gone nowhere. There doesn't seem to be any sort of interest in changing or anything like that, where there's particular patients where you go, "What's the point? I don't think I'm ever going to be able to make a change for this person" (Physiotherapist; outpatient care) - Definitely motivation is a huge factor and you can instantly tell whether someone is going to [take advice]... which isn't great because obviously the people that aren't motivated probably need it more than ever. But I guess you would gravitate less to those individuals (Physiotherapist; inpatient care). |
| Clinicians rely on their professional and interpersonal skills and exposure to appropriate training to promote NTPA | - I struggle with that [motivating patients] sometimes. Its very person dependent, got to try and find what's meaningful for them. I often try to utilise goals or use goals I should say, and try and sit down and go, what do you want to achieve in this time frame, this time frame, this time, right? Have multiple timeframes, and then try and use that as a motivating factor " (Physiotherapist; outpatient care) - We did do motivational interviewing at uni. But so that was very brief. So I've gone into I've gone and done further PDS into that to learn those skills, but also like I said in the first part when you're in grade one or new grad, those are skills that you often don't focus on because you're trying to get your physical skills going. So you often lose a lot of those motivational interviewing skills and need to re address them a little bit later in your career to up skill again in that (AEP; outpatient care) - They have to be motivating. You have to give someone a reason to want to do it. You can't just tell someone to do something if they don't have an interest (Physiotherapist; inpatient care) - Motivational interviewing, definitely do not utilize it enough. We definitely learned that in our undergraduate degree, but I wouldn't say I'm doing it to its truest form. I may be utilizing some components of it, but I definitely am not doing QR motivational interviewing (Physiotherapist; inpatient care) - I've used things like activity diary so then like actually writing it down and being accountable and so that's where, but that's for particular kind of people that that works with for (AEP; outpatient care) - Finding ways to relate to them. Like, whether it's like where they're from or you can even say things like, "Look, sometimes I don't want to exercise either, but afterwards if you're so good and like all these things improve, you're not always going to have the motivation, like you can't expect a way to come up with the motivation" (Physiotherapist; inpatient care) - If someone is motivated and completely ready to get to work, and wants to do things, and highly motivated, and comes across that way, then I'd say you're spending less time on educating them about the benefits, and actually just putting into practice, and guiding them with what they can do, versus if someone's not ready to change, there's evidence there if you've asked them to go away and do something and they haven't done it. Then there's no point flogging a dead horse and trying to keep asking them to do things (Physiotherapist; outpatient care) - Nothing specific that I look at, it's more getting a feel for person, so very non-specific and subjective for me as a clinician than anything (Physiotherapist; outpatient care) |

AEP: Accredited exercise physiologist; NTPA: Non-treatment physical activity
