# Supplementary material for: The promotion of non-treatment physical activity in physiotherapy and exercise physiology practice in an Australian regional hospital: A mixed-methods study
Source: JSAMS Plus. 2023 Jan 16;2:100020. doi: 10.1016/j.jsampl.2023.100020 (PMC13008451; doi:10.1016/j.jsampl.2023.100020)
Supplement: Multimedia component 7 [file mmc7.docx]

Supplement G. Criteria for rigor in study design

| **Criteria** | **How the research team fulfilled the criteria** |
| --- | --- |
| Worthy topic | - Determined as an issue that resonates with physiotherapists and AEPs practicing in public hospital settings – conception for study came from health promotion steering group of the physiotherapy/AEP department of the hospital - Determined as a robust way of gathering data on practical implications for clinical practice - In-depth literature review preceded the study |
| Rich rigor | - Interview questions designed from the findings of a clinician survey - Interviewed a wide range of physiotherapists and AEPs working across differing sectors of public hospital care - Use of concurrent analysis supplemented by field notes taken throughout interviews to continually develop key concepts and questions |
| Credibility | - Research team spanned individuals with 10+ years in physiotherapy/AEP practice, physical activity and public health research. - Consultation with stakeholders when developing interview guide - Inclusion of academic physiotherapist in research team with a PhD in the topic of non-treatment physical activity |
| Significant contribution | - The current research expands upon previous studies by examining rates and beliefs about NTPA promotion by physiotherapists and AEPs practicing in public hospital settings. - Recommendations for practice and policy are guided by established behaviour change framework (COM-B model) |

AEP: Accredited exercise physiologist; NTPA: Non-treatment physical activity
